# *Cyclospora cayetanensis*: A Perspective (2020–2023) with Emphasis on Epidemiology and Detection Methods

**DOI:** 10.3390/microorganisms11092171

**Published:** 2023-08-28

**Authors:** Sonia Almeria, Leonor Chacin-Bonilla, Jenny G. Maloney, Monica Santin

**Affiliations:** 1 Center for Food Safety and Nutrition (CFSAN), Department of Health and Human Services, Food and Drug Administration, Office of Applied Research and Safety Assessment (OARSA), Division of Virulence Assessment, Laurel, MD 20708, USA; 2 Institute of Clinical Research, University of Zulia, Maracaibo 4001-A, Venezuela; leonorbonilla42@yahoo.com; 3 Environmental Microbial and Food Safety Laboratory, Agricultural Research Service, United States Department of Agriculture, Beltsville, MD 20705, USA; jenny.maloney@usda.gov

**Keywords:** *Cyclospora cayetanensis*, epidemiology, food safety, methodology, outbreaks, review

## Abstract

*Cyclospora cayetanensis* infections are prevalent worldwide, and the parasite has become a major public health and food safety concern. Although important efforts have been dedicated to advance toward preventing and reducing incidences of cyclosporiasis, there are still several knowledge gaps that hamper the implementation of effective measures to prevent the contamination of produce and water with *Cyclospora* oocysts. Some of these data gaps can be attributed to the fact that access to oocysts is a limiting factor in *C. cayetanensis* research. There are no animal models or in vivo or in vitro culture systems to propagate the oocysts needed to facilitate *C. cayetanensis* research. Thus, researchers must rely upon limited supplies of oocysts obtained from naturally infected human patients considerably restricting what can be learnt about this parasite. Despite the limited supply of *C. cayetanensis* oocysts, several important advances have happened in the past 3 years. Great progress has been made in the *Cyclospora* field in the areas of molecular characterization of strains and species, generation of genomes, and development of novel detection methods. This comprehensive perspective summarizes research published from 2020 to 2023 and evaluates what we have learnt and identifies those aspects in which further research is needed.

## 1. Background

*Cyclospora* spp. are protozoan parasites of the phylum Apicomplexa that parasitize many species of mammals with remarkable host specificity [1,2,3]. Until recently, *Cyclospora cayetanensis* was the only species designated within the genus known to infect humans [4]. Several *Cyclospora* species have been found in other animals [3,5], and two new species have been described recently (*Cyclospora duszynskii* and *Cyclospora yatesi*) from Eastern moles (*Scalopus aquaticus*) in Arkansas, USA, based on distinct microscopical features of their oocysts [6].

*Cyclospora cayetanensis* has a direct life cycle and is transmitted between humans with no known intermediate or reservoir hosts. Infected humans shed unsporulated oocysts that are not infectious upon excretion. To become infectious, oocysts must undergo sporulation, which requires approximately 7 to 14 days under appropriate temperature and humidity conditions. The conditions required to facilitate sporulation have only been explored in laboratory settings to date [7,8]. Infection with *C. cayetanensis* occurs following ingestion of food or water contaminated with the sporulated oocysts and causes intestinal illness, characterized by explosive diarrhea, vomiting, fatigue, and weight loss [2].

*Cyclospora cayetanensis* infections are prevalent worldwide, and the parasite has become a major public health and food safety concern. In the United States (US), outbreaks of cyclosporiasis have been documented since the 1990s and have affected thousands of individuals annually in the past decade. Fresh produce appears to be an important vehicle for the transmission of *C. cayetanensis*, and outbreaks have been linked, among others, to ingestion of berries, cilantro, basil, and, more recently, ready-to-eat bagged salads [2,9,10,11]. Historically, cases were mainly a result of the consumption of imported fresh produce. However, in recent years, the US has had an increase in cases attributed to domestically grown commodities. Thus, there has been increased interest in testing fresh produce for the presence of *C. cayetanensis.* Several efforts have been put in place to develop microbiological detection methods for the parasite in both food and environmental water. These methods have been used to assist epidemiological investigations and survey the prevalence of *C. cayetanensis* in several commodities and growing regions. Despite these efforts, there are still several knowledge gaps that hamper the implementation of effective measures to prevent the contamination of produce with *Cyclospora* oocysts [2].

The biology, epidemiology, outbreaks, diagnosis, treatment, prevention, and control of *C. cayetanensis* have been summarized in previous reviews [1,2,12,13]. These reviews have outlined the many underdeveloped areas of *C. cayetanensis* research and highlighted future research needs. For example, until recently, details of the development of *C. cayetanensis* within the host were lacking, and there is still little information on the infective dose. Data demonstrating when sporulation takes place, the conditions that enable oocyst persistence in the environment, the role of water and soil in transmission, and the existence of potential reservoirs are all needed to implement control measures and limit spread. Additional studies on prevalence and population structure are needed to advance epidemiology, and there has been an urgent need for an effective genotyping method for source tracking in outbreak investigations.

Some of these data gaps can be attributed to the fact that access to oocysts is a limiting factor in *C. cayetanensis* research. There are no animal models or in vivo or in vitro culture systems to propagate the oocysts needed to facilitate *C. cayetanensis* research. Thus, researchers must rely upon limited supplies of oocysts obtained from naturally infected human patients, considerably restricting what can be learnt about this parasite. Despite the limited supply of *C. cayetanensis* oocysts, several important advances have happened in the past 3 years. Great progress has been made in the *Cyclospora* field in the areas of molecular characterization of strains and species, generation of genomes, and development of novel detection methods [14]. This comprehensive perspective summarizes research published from 2020 to 2023 and evaluates what we have learnt and identifies those aspects in which further research is needed.

## 2. Morphology and Life Cycle

Until recently, there were few studies on the life cycle of *C. cayetanensis*, and the existing information on its endogenous development was derived from histological examination of only a few biopsy specimens [15,16]. Although some of those reports included electron microscopic examination (TEM), the description of all life cycle stages was not completed. Recently, an extensive morphologic study using TEM provided features of the developmental stages of *C. cayetanensis* in the gallbladder of a man with human immunodeficiency virus [15]. Profuse multiplication of schizonts and the development of merozoites by schizogony were confirmed, and sexual stages were described in detail. However, type II long-sized merozoites were not found. The occurrence of merozoites of different sizes suggested the existence of multiple generations of schizonts. The study also demonstrated for the first time that the microgametes of *C. cayetanensis* have flagella.

Another TEM study using small intestinal tissue obtained from an immune-competent patient provided a further detailed description and confirmed the notably small endogenous stages (merozoites and gamonts) in *C. cayetanensis* [17]. The first analysis of the biopsy from the small bowel of this patient disclosed villous blunting and lamina propria inflammation, and high magnification revealed intracellular organisms in multiple life stages in the apical enterocytes consistent with *C. cayetanensis* [18]. The diagnosis of *C. cayetanensis* was confirmed by a multiplex PCR. TEM examination confirmed the presence of asexual forms (schizonts) and sexual forms (gamonts) within enterocytes, including immature and mature schizonts, an immature male gamont, and a female gamont [17]. Merozoites were small (<5 μm × 1 μm) and contained two rhoptries, a subterminal nucleus, numerous micronemes, and amylopectin granules. These parasite stages were like those previously reported in the gallbladder of the immunocompromised patient [15], suggesting that the general life cycle stages are not altered by the immunosuppression of the host. Although these two TEM studies have notably advanced the knowledge of the life cycle of *C. cayetanensis*, it will be helpful to have future studies that include cytological smears of intestinal biopsies to definitively establish the dimensions of endogenous stages of this parasite [17].

Unsporulated oocysts of *C. cayetanensis* are excreted by infected humans in feces. Sporulation occurs in the environment, but there are many unanswered questions concerning the dissemination and survival of *C. cayetanensis* oocysts. Research exists on the experimental conditions in which *C. cayetanensis* oocysts survive [7,8]. Unfortunately, there is no new information on what triggers oocyst sporulation in field conditions. Additionally, little is known of the biochemical composition of *C. cayetanensis* oocysts and their ability to persist in different environments, but like other protozoa, they are thought to be environmentally resistant. Further studies need to be performed in field conditions to address these questions but are hampered by the lack of a viability test and the limited availability of oocysts. Oocysts from other protozoa may represent a potential tool for addressing some of these questions. It has been proposed that working with other protozoans, such as *Eimeria*, *Toxoplasma,* and/or *Cryptosporidium*, could promote mitigation strategies for the removal of coccidian oocysts from irrigation waters [14].

## 3. Taxonomy

*Cyclospora* spp. belong to the phylum Apicomplexa, family Eimeriidae [2]. *Cyclospora cayetanensis* was first described in 1994 [19], and until very recently, it has been considered the only species of the genus *Cyclospora* known to infect humans [2].

A recent study that included the genotyping of thousands *Cyclospora* isolates from the US and one isolate from China [4] concluded that at least three genetic lineages of *Cyclospora* were responsible for human cyclosporiasis. Lineages A and B were found in specimens obtained in seasonal cyclosporiasis outbreaks in North America, while lineage C was represented by the *Cyclospora* isolate obtained from Henan Province, China. An assessment of heterozygous genotypes indicated a lack of gene flow between lineages A and B. A retrospective examination of epidemiologic data demonstrated associations between lineage and the geographical and temporal distribution of US infections. Additionally, the data supported that the gametes in these lineages are unable to produce a viable zygote post-fusion or that their gametes are unable to fuse. If this were not the case, vastly more mixed-lineage infections would have been observed. According to the authors [4], “lineages A, B, and C are in the nascent stage of speciation, where gene flow is highly disrupted (or absent), in support of reproductive isolation”. The authors provided an updated taxonomic description of *C. cayetanensis* (lineage A strains) and proposed two novel species as etiological agents of human cyclosporiasis: *Cyclospora ashfordi* (lineage B strain) and *Cyclospora henanensis* (lineage C strain) [4].

Lineages A and B seem to cause infections in the US at different times of the year and tend to also concentrate in different regions of the country, which could reflect the fact that the primary sources of foods imported into the US differ by region at different times of the year, suggesting that prior geographic isolation led to the recent divergence of lineages A and B [4]. Further research needs to be performed to ascertain the significance of these lineages/species from a clinical and public health perspective, whether certain produce items are more likely to be associated with lineage A or B, and/or whether lineage A or B is more virulent, more transmissible, or more likely to cause outbreaks [4]. The fact that measurements of unsporulated oocysts of lineage A and B showed overlapping features [4] and that to our knowledge, not differences at the clinical level have been observed among these lineages, makes these species distinctions preliminary.

## 4. Epidemiology

The attempts to characterize prevalence (in soils, water, foods, people) have generally been restricted to local/regional examination using a variety of diagnostic tools. Such methodological variation impairs attempts to compare studies as well as to discern more general patterns.

### 4.1. Human Prevalence

*Cyclospora cayetanensis* infection has been reported worldwide, in both developed and developing countries, with previous estimates of a global average prevalence of 3.5% [2,20,21]. Cyclosporiasis is endemic in most tropical countries, with large-scale epidemiological community-based studies reporting infection rates ranging from 0% to 41.6% [11]. In low-resource endemic areas with limited or poor hygiene standards, foreigners, children, the elderly, and immunocompromised individuals are the ones predominantly diagnosed with cyclosporiasis, while in non-endemic areas, people are affected without relevant effects of sex or age [2]. Infections with *C. cayetanensis* in pediatric populations in endemic countries are mainly diagnosed in children younger than 10 years of age [22].

Recent studies have shown high prevalence in poor rural communities in Venezuela (9.9%; 73/732) by microscopy [22,23], in indigenous people living in a remote region of tropical Colombia (11.8%; 16/136) by real-time PCR [24], and in HIV-positive individuals in Ghana (8.7%; 56/640) by real-time PCR [25]. On the other hand, lower prevalence of cyclosporiasis has recently been reported in several studies conducted in developing countries. For example, *Cyclospora* was only identified in 1.2%, 0.8%, and 0.4% of the 10,938, 133, and 60,501 samples examined in studies conducted in a hospital-based population in Honduras [26], in symptomatic patients in Cuba [27], and in patients submitting stools to a parasitology laboratory in Turkey [28], respectively. Notably, stool parasitological examination from a retrospective analysis of samples obtained during the pandemic (initial lock-down and gradual normalization periods) at the Diagnostic Parasitology Laboratory of Ege University (Izmir, Turkey) showed that the incidence of *Cyclospora* increased during the pandemic and gradual normalization periods [29]. However, due to the low number of samples, these differences should be taken with caution.

Prevalence varies by study, and variations in prevalence levels have been observed in the same country. In China, low prevalence of cyclosporiasis (0.2%, 13/5341) was observed among patients with diarrheal illnesses under surveillance at hospitals in Shanghai Municipality, Zhenjiang City, and Danyang City [30] and at Ningbo in Southeast China (0.61%, 3/489) [31], while a higher prevalence (10.0%; 5/50) was reported in a study in Yongfu country, also in Southeast China, that included the examination of samples obtained from facility workers in a laboratory animal facility with macaques (16.0%; 4/25) as well as from villagers nearby (4.0%; 1/25) [32]. In Iraq, a high prevalence of *Cyclospora* (12.1%; 68/560) was reported in patients attending hospitals and health centers in three cities, with rates of infection higher in the rural areas (14.5%) compared to the urban areas (8.4%) [33], while in another study in this country, which included patients attending outpatient clinics at two different hospitals in the capital Baghdad, *C. cayetanensis* was detected in 2.8% (1/229) of the examined patients in one hospital and <1.0% (1/271) in the second hospital [34].

A recent study in Gabon that examined stool specimens of children younger than 5 years old with diarrhea identified *C. cayetanensis* in 3.3% (8/241) of the samples analyzed using multiplex real-time PCR [35]. Similar prevalence (2.7%; 4/150) was observed in Ghana in samples collected from asymptomatic children under 5 years using microscopy [36]. In Iraq, the highest rate of infection was observed in children 1–9 years of age (25.8%; 29/112), which was significantly higher than rates observed in other age groups: 10–19 (21.8%; 21/96), 20–29 (16.6%; 12/72), 30–39 (1.0%; 1/93), 40–49 (3.6%;1/83), 50–59 (1.4%; 1/71), and 60–69 (3.0%; 1/33) [33]. However, in Thailand, *C. cayetanensis* was not observed using molecular detection in any of the 254 stool samples collected from school children in Ratchaburi Province at the Thailand–Myanmar border [37]. A comparison of stool detection methods for gastrointestinal viral, bacterial, and protozoan pathogens, including *Cyclospora*, was performed in stool samples from 632 Tanzanian children younger than 5 years of age, with and without gastrointestinal symptoms, by real-time PCR [38]. The number of samples positive for *C. cayetanensis* was four (0.9%), five (1.1%), and seven (1.5%) using nucleic acid extracted directly from stool, Whatman papers, and flocked swabs, respectively. Cycle threshold values showed no significant differences based on the nucleic acid extraction strategy.

Immunosuppressed patients are more prone and vulnerable to infection by parasites in general and to *C. cayetanensis* in particular. A significantly higher prevalence in HIV-positive individuals 8.7% (56/640) than in HIV-negative individuals 1.2% (1/83) was reported in Ghana [25]. The prevalence reached 13.6% in patients with CD4+ T cell counts below 200 cells/µL. Weight loss and diarrheal disease were also significantly more frequent in patients infected with *C. cayetanensis* than in patients negative to the parasite. Weight loss and diarrhea were reported in 36.4% and 20% of the *Cyclospora*-positive patients, while only 22.6% and 4.9% of the *Cyclospora*-negative patients reported those clinical symptoms. In Northeast China [39], it was observed that 1.6% of cases of *C. cayetanensis* were in HIV-positive men (7/384), whereas no positive cases were identified in the HIV-negative men examined (n = 199). According to a recent review of studies conducted on people living with HIV, the pooled prevalence of *C. cayetanensis* infection was 3.9% [40]. As expected, the prevalence of *C. cayetanensis* was higher in HIV-positive individuals than in healthy individuals, and there was a relationship of higher prevalence of cyclosporiasis when CD4 cell count was below 200 cells/µL and in those with diarrhea. Additionally, geographical differences in prevalence among immunosuppressed patients were identified, with the highest prevalence of *C. cayetanensis* infection reported in South America (7.9%) and the lowest in Asia (2.8%) [40].

A study that evaluated the occurrence of pathogens causing acute gastrointestinal infections from 2016 to October 2018 using data obtained by BioFire FilmArray Gastrointestinal Panel (Biomerieux, Inc. Salt Lake City, UT, USA), through the cloud database BioFire Syndromic Trends in samples (n = 91,401) from the US identified only 201 *Cyclospora*-positive samples (0.2%) [41]. Seasonality in the US was observed, with most cases concentrating between the months of March and September, with increased percentages around July.

Multiple risk factors have been commonly associated with the increased likelihood of contracting cyclosporiasis [2,22,23,39,40]. In recent Venezuelan studies, the most striking finding was the explicit association of *Cyclospora* infection with extreme poverty and soil transmission [22], confirming previous findings from a study in another community from the same country [42]. The link of cyclosporiasis with poverty carries relevant implications for targeted public health interventions in resource-poor cohorts. The high prevalence observed in a study in Iraq was attributed to the deterioration of the health and service conditions as well as the lack of clean drinking water, due to the war in the previous three years [33].

### 4.2. Outbreaks

Since the mid-1990s, *C. cayetanensis* has been recognized as the causative agent of multistate outbreaks of diarrheal illness in the US and Canada, with most outbreaks related to fresh produce consumption [2]. Similarly, several outbreaks have been reported in the past 5 years, mainly in the US and Canada (Table 1), mainly due to better detection methods and disease surveillance helping to track outbreaks [2].

In a case–control study of an outbreak in Canada, a total of 87 (1 hospitalized) locally acquired cases in four provinces from May to August 2016 were examined [43]. Although the study was not able to identify the exact source of the outbreak, case exposure identified some possible foods, with imported blackberries from Mexico being the most likely source of infection. In the US, a cyclosporiasis outbreak of diarrheal illness in June and July 2018 (two separated clusters) was reported, affecting two training populations in the US Air Force at a joint base in San Antonio–Lackland, Texas [44]. In cluster 1, 46 suspected and 7 confirmed cases occurred, while in cluster 2, 18 suspected and 14 confirmed cases were reported. The questionnaires showed related risk with blueberries, blackberries, cherry tomatoes, and oranges, but no source was conclusively identified. At the time of the outbreak, there were no known connections to the larger national outbreaks related to vegetable trays or salads from a restaurant chain that were contemporaneously occurring [44].

In Asia, an outbreak was reported in South Korean travelers returning from Nepal that constituted the first outbreak of cyclosporiasis in the country [45]. The outbreak included eighteen South Korean residents who traveled together to Nepal in 2013. One of the travelers developed chronic watery diarrhea after returning to South Korea and sought medical care. After this first consultation, additional stool samples were collected from seven additional travelers. Three out of eight were positive for *C. cayetanensis* infection by PCR, and sequencing confirmed the *C. cayetanensis* infection. The source of infection was believed to be local foods including vegetables, water, or fruits based on an epidemiological survey of the patients.

**Table 1:** *Cyclospora cayetanensis* outbreaks published between 2019 and 2022 *.

Country (Sites)	Year	No. of Cases (No. of Laboratory Confirmed Cases)	Suspected Source (If Known, Cases Related to the Produce)	Origin/Notes	References
Canada (4 provinces)	2016	87	Blackberries	Mexico	[43]
Republic of Korea	2020	8 (3)	Vegetables, water, or fruits	Nepal	[45]
USA, Texas	2018	Cluster 1: 46 (7) Cluster 2: 18 (14)	Blueberries, blackberries, cherry tomatoes, and oranges	Unknown	[44]
USA (37 states)	2019	2408 (2408)	Basil (241)	Mexico	[46]
USA (35 states)	2020	1241 (1241)	Bagged salad mix containing carrots, red cabbage, and iceberg lettuce (701)	USA	[47]
USA (36 states)	2021	1020 (1020)	Leafy greens	Unknown	[48]
USA (33 states)	2022	1129 (1129)	Leafy greens	Unknown	[49]

* For outbreaks reported before 2019, see Almeria et al. (2019) [2].

In the USA, herbs and particularly leafy greens have been the most frequent produce linked to recent outbreaks (Table 1). Of interest is the fact that *C. cayetanensis* contamination has been reported in domestically grown produce (cilantro and romaine lettuce) in recent years.

### 4.3. Seasonality

*Cyclospora cayetanensis* infection is remarkably seasonal worldwide. Seasonality varies by region, most likely due to human activities, environmental contamination, and the optimal sporulation conditions in each area. The seasonal trends in the epidemiology of cyclosporiasis are inconsistently reported over different geographic regions [2]. Cyclosporiasis often coincides with warm periods of maximal rainfall, as reported in Mexico, Guatemala, Honduras, Colombia, Cuba, Jordan, Nepal, Indonesia, and China. By contrast, infection is more prevalent during the dry season in countries like Peru and Turkey, while it increases during colder months in Haiti [2,22]. Therefore, environmental factors such as rainfall, temperature, and humidity seem to be important in the parasite life cycle, but the effect of environmental conditions on parasite survival and transmission remains unclear [22].

Recent studies in endemic areas have added information on seasonality. In a study conducted in Venezuela, *Cyclospora* prevalence was 9.9% (73/732), with monthly variation from 0% to 35.3% [22]. The fluctuation of infections was associated with rainfall, with an increase in infections during months with more rainfall with a bimodal distribution for the infection. The first peak occurred in June (24.0%), and there was a second narrow and steep peak (35.3%) in October when the highest rainfall was noted. The average monthly mean temperature during the study had low variability (26.4 to 29 °C) and a change in infection rate related to this variable was not observed. This study constitutes the first evidence of the seasonality of *Cyclospora* in infected people in Venezuela, providing important insights into its epidemiology [22]. In another study in South America, an increased rate of *C. cayetanensis* infections was observed in the rainy season (July–November) in a Colombian Indigenous Wiwa population [24] when compared to results reported in the same area in the dry season (January–April) [50]. The two studies were performed four years apart [24,50]. After the first assessment that identified a high baseline of *C. cayetanensis* infection in the rainy season [50], a second epidemiological follow-up assessment in the dry season was conducted. Higher prevalence and higher parasite loads (associated with lower Ct) were observed in the rainy season (11.8%, 16/136; Ct 30.6 ± 3.4) compared to the dry season (5.1%, 15/292; Ct 34.4 ± 1.6), respectively. However, fewer individuals (2/16, 12.5%) reported gastrointestinal symptoms in the rainy season compared to the dry season (6/15, 40%). Seasonality is observed on other continents as well. Among patients attending health centers in three cities in Iraq, the highest rate of infection was recorded at 41.5% in April and the lowest at 2.5% in November; no infections were recorded during the summer [33].

As indicated above, in the US, clear seasonality of *Cyclospora* infections has been observed, with most cases concentrating between the months of March and September, with increased percentages around July [41].

There is a need for more studies from diverse regions/countries to assist in understanding the determinants of seasonality. Furthermore, the reasons for the apparent absence of symptomatic human infection for prolonged periods when the parasite is still present in the environment, and what biological conditions are needed for the survival of the parasites during prolonged periods in the environment, remain unknown [2,51]. One hypothesis is that hypobiosis, or arrested development, may occur either inside or outside the host according to the climatic environmental conditions and seasonality experienced by the parasite and/or host in a particular region. This is a well-established occurrence for some parasites, such as gastrointestinal nematodes or bot flies (i.e., *Caenorhabditis elegans*, *Strongyloides stercolaris*, *Ostertagia ostertagi*, *Cooperia oncophora*, or *Oestrus ovis*) [52,53,54]. These parasites stay in hypobiosis until the appropriate triggers initiate replication. This highly coordinated process ensures that infective forms enter the environment when the weather conditions are optimal for survival and transmission to new hosts. Similarly, *C. cayetanensis* could sense environmental conditions and only mature when the conditions are conducive for survival; something besides rainfall could trigger sporulation. To explain the timing, *C. cayetanensis* would need to mature ahead of the rainy season. Data from surveillance in Canada and the US have shown peaks in the percentage of produce samples that were positive for *C. cayetanensis* after the outbreak season (unpublished data). If *Cyclospora* follows a hypobiosis phenomenon, people could become infected by consumption of the parasite in produce well ahead of the *C. cayetanensis* outbreak season. Then, the parasite would remain dormant in the intestine of infected persons and only start multiplying and producing symptoms around May–June (in the US), when environmental conditions that promote transmission are optimal. A major limitation to studying and confirming this hypothesis is that the required analysis and enumeration of the parasitic merogony and schizogony stages in the human intestine would be hindered by the obvious ethics issues related to any study that involves using intestinal tissues from humans.

### 4.4. Cyclospora spp. in Food

Contaminated fresh produce has long been recognized as an important source of infection by *C. cayetanensis*. Beginning in the 1990s, fruits and vegetables, mainly berries, herbs, and leafy greens, were linked to foodborne *C. cayetanensis* clinical cases and outbreaks in the US [1,2]. Such observations have spurred an interest in understanding the role of fresh produce in the transmission of *C. cayetanensis*.

*Cyclospora cayetanensis* contamination has been reported in fresh produce surveillance studies in several countries, mainly in endemic areas [2]. Several studies have been performed in recent years that added data on the presence of *Cyclospora* spp. and/or *C. cayetanensis* in fresh vegetables and fruits (Table 2). In a recent systematic review and meta-analysis of the global prevalence of intestinal protozoan parasites in vegetables and fruits, it was found that the pooled prevalence of *C. cayetanensis* in vegetable samples was 6% but with large heterogeneity among surveys [55].

**Table 2:** Prevalence of *Cyclospora* spp. or *C. cayetanensis* in fresh produce worldwide (since 2019).

Country	% (No. Positive/Total Samples Analyzed)	Food Type	Method	References
Canada	0.28% (5/1759) imported leafy green, herb, and berry samples. *C. cayetanensis* was detected in berries (two), herbs (two), and leafy greens (one)	Imported leafy green, herb, and berry samples	BAM chapter 19b implemented in the CFIA (qPCR)	[56]
China (Henan) Agricultural farms and open markets	Total 0.3% (3/1120): 0.2% in vegetables (2/1099) and 4.8% in fruits (1/21). Vegetables: 0.5% lettuce (1/200) and 2.3% in leaf lettuce (1/44). Two samples confirmed by sequencing	Lettuce, coriander, celery, baby bok choy, leaf lettuce, water spinach, crown daisy, fennel plant, endive, spinach, schizonepeta, cabbage, leaf mustard, Chinese chive, and chive, and the stripped epidermis of bacca (cucumber, watermelon, potato, bean, green chili).	Nested PCR and sequencing	[57]
Colombia (Bogota) Retail markets in 20 localities	0.83% strawberries (1/120)	Strawberries	Multiplex quantitative PCR (qPCR) assay	[58]
Egypt (Assuit City, Assuit governorate)	Total 2.9% (7/240): 7.5% watercress (3/40), 5.0% parsley (2/40), 2.5% radish (1/40), and 2.5% green onion (1/40). Negative coriander (0/40) and lettuce (0/40)	Watercress, radish, parsley, coriander, green onion, and lettuce	Flotation and sedimentation. Lactophenol cotton blue stain and Modified Ziehl–Neelsen stain	[59]
Egypt (El-Kharga Oasis, Upper Egypt) from public markets	Total 20% (54/270): 20% arugula (*Eruca sativa*) (36/180) and 20% radish (*Raphanus sativus*) (18/90)	Arugula and radish	Sedimentation and flotation techniques with modified Ziehl–Neelsen staining	[60]
Ethiopia (Arba Minch town, southern Ethiopia)	Total 2% (7/347): 4% tomatoes (4/100), 3% green peppers (2/66), and 4.3% salad (1/23). 0% carrots (0/62), 0% cabbage (0/96)	Tomatoes, cabbage, green peppers, carrots, salads	100 g sample. Light microscope with Lugol’s iodine and Modified Ziehl–Neelsen staining	[61]
Ethiopia (Nine local markets in Dire Dawa City)	Total 7.4% (28/376): 17.9% lettuce (5/47), 7.1% cabbage (2/47), 10.7% carrots (3/47), 14.3% tomato (4/47), 14.3% green pepper (4/47), 17.9% banana (5/47), 3.6% orange (1/47), 14.3% spinach (4/47)	Lettuce, cabbage, carrots, tomato, green pepper, banana, orange spinach	Sedimentation. Modified Ziehl–Neelsen staining	[62]
Iraq (Baghdad), local markets	Total 3.7% (2/54): 5.5% lettuce (1/18), 5.5% basil (1/18), 0% parsley (0/18).	Lettuce, parsley, and basil	Five-hundred-gram sample size. Light microscope	[34]
Iraq (Anbar province)	No data on number of samples collected from each vegetable Counted oocysts from each produce were 6, 7.8, 7.2, 4.4, and 3.2 oocysts/liter washing of garden cress, radish, leek, green onions, and purslane, respectively.	Garden cress, radish, leek, green onions, and purslane	Saturated sugar, MO, fluorescent microscope, and counting in Newbauer chamber slide	[33]
Italy (Apulia), supermarkets in Foggia and Bari towns	Total 0.08% (1/1296): 0.3% blueberries (1/324 pools), 0% ready-to-eat (RTE) mixed salad (0/324), 0% blackberries (0/324), 0% raspberries (0/324)	RTE mixed salads (locally produced), blueberries and blackberries imported from Peru and Mexico, respectively, and raspberries grown in Italy	Saturated zinc sulfate solution. Confirmation by three molecular PCRs (BAM qPCR, other qPCR, and nested PCR) and sequencing	[63]
Mexico (commercial farms in central Mexico)	Total 23.5% (4/17): 16.6% blueberries (1/6) and 27.3% blackberries (3/11)	Blueberries, blackberries	Nested PCR assay, confirmation by Sanger DNA sequencing and phylogenetic analysis	[64]
Mexico (Caborca, Northwest Mexico). Ten open markets and three packing centers	Total 2.7% (4/150): 6% in open-air markets (3/50) and 2% (1/50) from substandard asparagus bundles, 0% (0/50) for exportation	Asparagus	Kinyoun staining	[65]
Mexico (Caborca, Northwest Mexico); unregulated (8) and regulated (8) open-air markets, agricultural field for export produce	Total 11.0% (44/400): 1% melon (1/100), 5% peach (5/100), 8% asparagus (8/100), 30% grapes (30/100)	Melon, peach, asparagus, and grapes	Kinyoun staining	[66]
Norway Markets	Total: 6.6% (52/820): 5.5% blueberries (15/274), 8.7% raspberries (24/276), and 4.8% strawberries (3/270)	Blueberries, raspberries, and strawberries	Multiplex qPCR	[67]
Venezuela (Falcon State)	Total 3.9% (3/77) of 5 units of lettuce, cabbage, celery, cilantro, and parsley; 8 units of peppers, tomatoes, onions, and mushrooms; and 20 units of strawberries	Lettuce, cabbage, celery, cilantro, parsley, peppers, tomatoes, onions mushrooms, and strawberries	Concentration and UV epifluorescence and phase-contrast microscopy	[22]

At the Canadian Food Inspection Agency (CFIA) laboratory, the BAM chapter 19b method using a new detection platform (Bio-Rad CFX96, Bio-Rad, Hercules, CA, USA) was used to examine 1759 imported leafy green, herb, and berry samples for the presence of *C. cayetanensis* [56]. The parasite was detected in berries (two), herbs (two), and leafy greens (one), representing 0.3% of the tested survey samples. In Italy, a survey that included 324 fresh produce samples collected at markets and used microscopy following concentration of parasite forms using zinc sulfate identified *Cyclospora*-like oocysts in a blueberry sample (imported from Peru) [63]. This finding was later confirmed to be *C. cayetanensis* by three molecular methods: US BAM specific qPCR (Assay 1), multiplex qPCR for *Cyclospora* and other parasites (Assay 2), and nested PCR coupled with sequencing (Assay 3) [63]. *Cyclospora cayetanensis* was also found in a surveillance study in berries sold in Norwegian markets [67]. The survey analyzed 820 berry samples, both imported and produced in Norway, including 274, 276, and 270 from blueberries, raspberries, and strawberries, respectively. The overall occurrence of *C. cayetanensis* in berries was 6.6% and *C. cayetanensis* was found in 8.7%, 5.5%, and 4.8% of the raspberries, blueberries, and strawberries, respectively. This study was the first to detect the parasite in berries in Norway since a previous survey of fresh produce samples in this country did not detect *C. cayetanensis* [59]. This may reflect that the potential for contamination has increased since the initial survey was conducted or that in the first study, microscopy was used to detect parasites versus molecular assays used in the more recent survey [67,68].

Two studies were recently conducted in fresh produce samples in two different provinces of Iraq. The first study was conducted in Baghdad Province. In that study, *Cyclospora* oocysts were detected in 3.7% of the 54 samples of fresh produce (lettuce, parsley, and basil) examined using light microscopy [34]. The second study in Anbar province examined washing water from five types of vegetable leaves (garden cress, radish, leek, green onions, and purslane) [33]. *Cyclospora* was found in at least one of the studied types of vegetables and the number of oocysts per liter was 6, 7.8, 7.2, 4.4, and 3.2 in garden cress, radish, leek, green onions, and purslane, respectively. In China, a survey conducted in the Henan province examined 1099 of a variety of vegetable and fruit samples obtained from agricultural farms or open markets to detect the presence of foodborne parasites *Cryptosporidium* spp., *Giardia duodenalis*, *C. cayetanensis*, and *Enterocytozoon bieneusi* using molecular methods [57]. *Cyclospora cayetanensis* was only identified in two vegetable samples (0.2%). PCR detection was followed up with sequencing to confirm that it was *C. cayetanensis*, nucleotide sequences were found to be identical to a nucleotide sequence obtained from a human isolate from Shanghai (KJ569533). In a study of Korean fresh-cut fruit products at retail, a total of 25 different types of fresh-cut fruit products were evaluated, but no *Cyclospora*-positives were found by real-time PCR [69].

In central Mexico, *C. cayetanensis* was detected by nested PCR in 16.6% (1/6) and 27.3% (3/11) of the blueberry and blackberry samples obtained from commercial farms, respectively. Sanger DNA sequencing and subsequent phylogenetic analysis confirmed the presence of *C. cayetanensis* in those berries [64]. In Caborca, Northwest Mexico, two different studies analyzed the presence of *Cyclospora* in fresh produce. In this area of Mexico, there is semi-warm and extremely dry weather almost year-round, with only 209 mm of rainfall annually. The first study was a cross-sectional study of fresh asparagus [65]. The authors analyzed 150 bundles of asparagus for export (50), sub-standard (50), and from open-air markets (50). The presence of *Cyclospora* spp. was observed in 3% of the samples by microscopy. In the second study, a total of 400 fruit and vegetable samples from unregulated open-air markets and closed markets including melon, peach, asparagus, and grapes as well as 100 bundles of asparagus from an agricultural field for export (50 export-grade and 50 sub-optimal) were examined [65]. *Cyclospora* spp. was found in 44 of the samples (11.0%), specifically in 1.0%, 5.0%, 8.0%, and 30.0% of the melon, peach, asparagus, and grape samples, respectively. No differences were observed in the percentage of positive samples between samples collected in open-air markets (11%; 22/200) and closed markets. A recent study of strawberry samples obtained at supermarkets and local markets was conducted using a multiplex qPCR assay in Bogota, Colombia [58]. Only one strawberry sample (0.8%; 1/120) from a local market was found positive for *C. cayetanensis*. In Venezuela, 3 out of 77 (3.9%) samples of fresh produce (peppers, tomatoes, onions, mushrooms, strawberries, lettuce, cabbage, celery, cilantro, and parsley) collected at local markets examined by microscopy were positive for *Cyclospora* [22].

In Southern Ethiopia, a survey examined 347 vegetable samples (tomatoes, cabbage, green peppers, carrots, salad) for the presence of *Cyclospora* spp. using microscopy [61]. *Cyclospora* spp. was detected in seven samples that included 4, 2, and 1 out of the 100, 66, and 23 tomatoes, green peppers, and salad samples examined, respectively. It was determined that vegetables directly supplied by farmers to vendors were more prone to parasitic contamination as compared to those supplied by large-scale vendors. Also in Ethiopia, another study identified the presence of *Cyclospora* spp. in eight types of fruits and vegetables (lettuce, cabbage, carrot, tomato, green pepper, banana, orange, and spinach) obtained from selected local markets in Dire Dawa City [62]. In this study, fruits/vegetables not washed before display were almost three times more likely to be contaminated with foodborne parasites than those washed before display. Similarly, means of display also have an impact on the odds of being positive for parasites, with being displayed on the floor/ground increasing the likelihood of being contaminated fivefold versus produce displayed on tables/shelves. This might be an indication that post-harvest contact with the floor could play a role in contaminating fresh produce. In Egypt, 240 samples (40 from each of watercress, radish, parsley, coriander, green onion, and lettuce) were examined by microscopy after simple washing [59]. *Cyclospora* spp. showed a prevalence rate of 2.9% (7/240), with positive samples observed on watercress (3/40), parsley (2/40), and radish and green onion (1/40), but the parasite was not detected on lettuce or coriander. In another study in this country, *Cyclospora* was reported in fresh produce cultivated in El-Kharga Oasis, Upper Egypt, that was obtained at public markets [60]. Of the samples of arugula (36/180) and radish (18/90) examined, 20% each were *Cyclospora*-positive by microscopic examination.

There are few reports of *Cyclospora* presence in other food matrices. A study observed the presence of *C. cayetanensis* in blue crabs in Europe [70]. The study molecularly investigated the hemolymph, gills, stomach, hepatopancreas, and gonads of eleven invasive Atlantic blue crabs (*Callinectes sapidus*) from the Lesina Lagoon (Mediterranean Sea) for the presence of the parasite. A high prevalence was observed, with 4 of the 11 Atlantic blue crabs examined being found positive for *C. cayetanensis* (36.4%). Of 55 tissue samples analyzed, 14.5% were positive for *C. cayetanensis*, with hemolymph and gills being the most infected tissues. A recent study reported the presence of *Cyclospora* spp. by microscopy in the digestive system of the lobster cockroach (*Nauphoeta cinerea*) (n = 32) in Brazil [71], but those results need confirmation by molecular methods.

Only two studies analyzed the presence of *Cyclospora* spp. in animals in recent years. In Baghdad, the presence of *Cyclospora* spp. oocysts was studied in fecal samples from 31 dogs, 19 cats, and 100 rats. Dogs and cats were negative. However, they observed using microscopy that 3% of 100 rats were passing *Cyclospora*-like oocysts. [34]. In China, in the Yunnan Province, the prevalence of *Cyclospora* spp. in cattle was 2.5% (13 of 524 fecal samples) analyzed by nested PCR and RFLP. Prevalence was not related to region (four regions studied), sex, or age. Phylogenetic analysis classified five *Cyclospora* spp. samples into the *C. cayetanensis* group, and the authors concluded that there could be zoonotic potential [72]. However, as indicated earlier [2], the presence of oocysts in animals may simply reflect passage through the gastrointestinal tract, since to date there is no evidence of tissue infection in animals. Therefore, further confirmation of infection in animals is needed to confirm any zoonotic potential. On the other hand, the possibility that animals could aid in the dissemination of oocysts in the environment cannot be dismissed.

### 4.5. Cyclospora spp. in Water

*Cyclospora cayetanensis* oocysts have been detected in several types of water [2,73,74], including chlorinated water and wastewater in endemic and non-endemic areas, suggesting water as a vehicle of transmission. The pooled prevalence of *Cyclospora* in water has been estimated to be 6.9% globally [75]. Additionally, it was reported that of the seven different water sources considered, irrigation water had the highest prevalence of 17.1%, nearly triple the prevalence reported in recreational, surface, and drinking waters, although it is important to note that differences were not statistically significant [75]. Thus, in addition to contributing to human infection through direct consumption, water is likely an important vehicle for indirect transmission through the application of contaminated irrigation water to produce intended for raw consumption. There have been recent findings of *C. cayetanensis* in wastewater, some watersheds, and other sources of water, particularly agricultural water [22,76,77,78,79,80,81,82,83], that reinforce the contribution of humans to water contamination. The fact that contaminated water might be used for irrigation of produce and, therefore, contaminate produce, supports the importance of future studies focused on understanding the role of agricultural water in the transmission of the parasite.

In Northwestern Venezuela, using UV epifluorescence and phase-contrast microscopy, 4/14 (28.6%) water samples were *Cyclospora*-positive. Of those samples, 2/10 (20%) were water wells, 1/2 (50%) from water trucks, and 1/2 (50%) from river water [22]. A recent large-scale study [79] performed molecular characterization of several waterborne pathogens (*Cryptosporidium* spp., *Giardia duodenalis*, *Enterocytozoon bieneusi*, *C. cayetanensis* and *Eimeria* spp.) in wastewater and sewage in Guangzhou, China. A total of 238 influent samples were collected from four wastewater treatment plants (WWTPs) along with samples from eight sewer locations. *Eimeria* spp. and *Cyclospora* spp. were detected using a common nested PCR (294 bp fragment of the *SSU* rRNA gene) and then were individually identified by sequence analysis of the secondary PCR products. *Cyclospora cayetanensis* was detected in three sewer samples and one WWTP sample (total prevalence: 4/150; 2.7%). Although the detection of *C. cayetanensis* in wastewater samples was sporadic, its presence has significant implications for the occurrence of cyclosporiasis in this population. Detection of *Cyclospora* spp. and *Cryptosporidium* sp. oocysts by microscopy, increased in a major Philippine watershed following rainfall events [81]. A study was carried out in the Mezam watershed in Bamenda, Northwest Region of Cameroon, in which direct concentration and the Ziehl–Neelsen technique were used for diagnosis [76]. Among other protozoa parasites, *C. cayetanensis* was found in a concentration of 141.31 ± 143.19 oocysts/l, and higher densities of oocysts were observed in the dry season (471.42 ± 216.32 oocysts/l). In Turkey, of 36 agricultural irrigation water samples collected in seven different stations in Denizli City Center, *Cyclospora* spp. was found in 5 samples (5.95%) by microscopy. The parasite was not found in drinking water (n = 48) [83].

### 4.6. Cyclospora spp. in Soil

Soil is a potential and possibly important mode of transmission and source of infection for *C. cayetanensis* [2,42,84,85]. The contamination of soils by inadequate defecation disposal might be a significant determinant for infection. Some studies have included contact with soil as a risk factor for *C. cayetanensis* infections, in both developing and developed countries [2,13]. Until recently, there was a dearth of publicly available detection-method studies in soil (n = 0) and water (n = 2) as well as of studies of prevalence in soil (n = 1) [86]. In recent years, some additional publications reported the presence and/or prevalence of *C. cayetanensis*-like oocysts and/or DNA in soil [22,64].

In Mexico, *C. cayetanensis* was detected in 20% (1/5) of soil samples obtained from Jalisco, Guanajuato, and Michoacán by nested PCR [64]. In Northwestern Venezuela, using UV epifluorescence and phase-contrast microscopy, 9/50 (18%) soil specimens were positive for *Cyclospora* spp. [23]. *Cyclospora* spp. infections predominated in the months of higher rainfall, which supports that mean annual rainfall and the consequent moisture of the soil helped in the survival and maintenance of oocysts in the soils of this semiarid region [22].

## 5. Detection Methods

### 5.1. Fresh Produce

Given the role of fresh produce as an important source of *C. cayetanensis,* there is a clear need for specific and sensitive methods that can detect this parasite in fresh produce samples to understand infection sources and improve public health. Testing fresh produce for the presence of *C. cayetanensis* can be a difficult process. The oocyst form of the parasite may be present in low concentrations, requiring methods that are sensitive. DNA extracts from fruits, vegetables, and prepared foods are complex matrices that may contain PCR inhibitors that produce false negative results. Produce samples may also contain other organisms closely related to *C. cayetanensis*, requiring detection methods with high specificity.

A recent review focused on the molecular methods for the detection of *C. cayetanensis* in fresh produce [87]. The detection of *C. cayetanensis* in fresh produce has been performed using several methods including fluorescence microscopy, conventional PCR, and TaqMan probe-based qPCR [87,88,89]. PCR-based methods for the detection of *C. cayetanensis* in produce have traditionally been performed using primers targeting the 18S rRNA gene, internal transcribed spacer 2 (ITS 2), or the hsp70 gene [87]. However, new targets have recently been developed for screening produce samples for the presence of *C. cayetanensis* [82,88,89,90]. Quantitative real-time PCR of the internal transcribed spacer 1 (ITS1) was developed and validated for use in berries [88]. This same primer set was also validated for use in a multiplex qPCR for the simultaneous detection of *C. cayetanensis*, *Echinococcus multilocularis*, and *Toxoplasma gondii* [89].

A conventional PCR method demonstrated that a region of the cytochrome oxidase gene could be used to confirm the presence of *C. cayetanensis* in samples screened using other methods. The amplicon produced using this method contained enough sequence-level discriminatory power to confirm the presence of *C. cayetanensis* in samples screened using the current FDA Bacteriological Analytical Manual (BAM) 19b method [82]. Additionally, the FDA recently developed and validated a refined and specific real-time PCR detection method using mitochondrial primers (Mit1C qPCR) for the detection of *C. cayetanensis* in produce [91].

Two recent studies further developed molecular methods for *C. cayetanensis* detection from fresh produce using nested PCR procedures that target the 18S rRNA gene. A multiplex PCR for *C. cayetanensis* was developed that simultaneously detects *C. cayetanensis* as well as *Cryptosporidium* spp., *Giardia* spp., and *Toxoplasma gondii* [92]. This multiplex assay employed a nested PCR strategy with custom primer sets for the SSU rRNA gene to achieve simultaneous detection and differentiation of *C. cayetanensis* from spinach samples [92]. Another study used a nested PCR approach targeting the SSU rRNA gene and previously reported primers and demonstrated that this strategy could be used to test for *C. cayetanensis* from berries [64].

When screening laboratories identify *C. cayetanensis*-positive fresh produce samples, tools for further analysis of positive samples to extract genetic information that can be used to distinguish isolates and identify potential contamination sources will be needed. Highly conserved genes may not represent good targets for achieving these goals as they are unlikely to contain the genetic diversity needed to distinguish differences among closely related isolates of *C. cayetanensis.* However genomic data and next-generation sequencing strategies represent potential tools for overcoming these limitations. Recently, two methods for obtaining the mitochondrial genome of *C. cayetanensis* from produce samples using Illumina sequencing (Illumina, Inc. San Diego, CA, USA) were published [90,93]. The first method used four primer sets that produced overlapping amplicons to cover the entire mitochondrial genome and demonstrated that this method could be used to sequence the mitochondrial genome of *C. cayetanensis* from cilantro samples [93]. The method was able to detect 200 oocysts in artificially contaminated cilantro samples. The second method utilized a custom AmpliSeq panel consisting of 35 primer sets to amplify and then sequence the whole mitochondrial genome [90], and it was able to detect as few as five oocysts in artificially contaminated produce samples. These methods were designed to complement the current validated FDA BAM 19b method for the detection of *C. cayetanensis* in fresh produce to allow for the genotyping of positive samples to enhance surveillance activities and outbreak investigations [90,93,94]. Another assay based on targeted amplicon sequencing (TAS) incorporating an enrichment step to gain the requisite sensitivity for genotyping *C. cayetanensis* contaminating fresh produce samples was recently reported [95]. This new TAS assay targets 52 loci (49 in the nuclear genome and the rest at the mitochondrial level) and encompasses 396 currently known SNP sites. The performance of the TAS assay was evaluated in lettuce, basil, cilantro, salad mix, and blackberries inoculated with *C. cayetanensis* oocysts. A minimum of 24 markers were haplotyped even at low contamination levels of 10 oocysts in 25 g leafy greens. Oocysts from two different sources were used for inoculation, and samples receiving the same oocyst preparation clustered together, but separately from the other group. This assay would allow for linking clinical and produce samples and represents a significant advance in the ability to genotype *C. cayetanensis* contaminating fresh produce [95].

New methods for the downstream analysis of positive samples are needed to enhance our ability to study the genetic differences among isolates to link positive samples with potential contamination sources. Leveraging emerging technologies based on whole genome sequence data and next-generation sequencing platforms may provide additional novel strategies for tackling the unique challenges associated with sampling fresh produce for *C. cayetanensis*.

Another important consideration for method development for the detection of *C. cayetanensis* from fresh produce is the validation of the detection method for the diverse groups of substrates that may require testing. Recently, the Canadian Food Inspection Agency (CFIA) laboratory independently verified the performance characteristics and robustness of the BAM chapter 19b method for the detection of *C. cayetanensis* in a variety of matrices, including under adverse sample conditions, using a new detection platform (Bio-Rad CFX96) [56]. They found that the diagnostic and analytical specificity were 100% for all matrices and related parasites tested and that the proportion of positive samples was unaffected (*p* = 0.22) by age or condition of produce (7 d, fresh, frozen) or wash concentrate (3 d, fresh, frozen). A novel duplex real-time PCR assay using primer/probe combination (Mit1C) targeting a conserved region of the mitochondrial genome and an internal amplification control (IAC) for the detection of *C. cayetanensis* in produce following the BAM chapter 19b method was validated in a single study [91]. The method was validated using three food matrices, cilantro, raspberries, and romaine lettuce, and demonstrated to be sensitive, detecting as few as five oocysts, and specific based on results from the inclusivity/exclusivity panel.

As methods are validated for use with different products it is likely that modifications will be needed to enhance the sensitivity and specificity of assays intended for sample screening. For example, sample washing, DNA extraction techniques, and choice of PCR master mix have all been shown to influence the successful detection of *C. cayetanensis* from produce samples [10,56,96]. The condition of the produce at the time of testing may also be an important consideration as the detection of *C. cayetanensis* from bagged salad before and after sell-by dates was shown to differ significantly [11]. Proper validation of screening methods may be product-dependent and is a necessary step in ensuring the suitability of a screening method for use with fresh produce.

### 5.2. Water

There is a clear need to understand the potential for different water sources to serve as points of infection for *C. cayetanensis*, but it is important to consider the distinct challenges associated with detecting *C. cayetanensis* in water. It is expected that the infectious stage of the parasite may be present in water at very low concentrations. Additionally, water is a complex substrate that may contain any variety of contaminating substances depending on its origin. Thus, the detection of *C. cayetanensis* in water may require concentration techniques that enrich and purify the oocyst form of the parasite to enhance detection. Following the concentration of *C. cayetanensis* oocysts from water samples, testing for the presence of *C. cayetanensis* is performed using microscopy, molecular methods, or both. The methods of water analysis must be able to overcome the unique challenges that come with working with water, such as the need to process large volumes of water of varying compositions. Techniques for *C. cayetanensis* detection must also be both sensitive and specific, as water may contain closely related organisms that are not human pathogens.

Historically, a variety of methods have been used to clean and concentrate *C. cayetanensis* oocysts from water. A recent systematic review and meta-analysis of the prevalence of *C. cayetanensis* in water that included 33 studies published between 1993 and 2019 reported concentration techniques including filtration, centrifugation, sedimentation, and flocculation, all used either alone or in combination in one or more of the studies included in the analysis [75]. Additionally, gradient centrifugation and sucrose flotation are also reported methods for cleaning and concentrating *C. cayetanensis* oocysts [80]. Although a variety of methods for sampling water for the presence of *C. cayetanensis* have been reported, studies with the expressed goal of developing detection methods specifically for water are rare [86]. In 2019, the FDA released an updated method for the detection of *C. cayetanensis* from agricultural water (BAM 19c) that uses quantitative PCR of a region of the 18S rRNA gene for the molecular detection of *C. cayetanensis* [78].

In recent years, there have been several studies on method development for water processing and *C. cayetanensis* detection [77,78,80,82]. A recent study demonstrated that dead-end ultrafiltration (DEUF) coupled with quantitative PCR could consistently detect *C. cayetanensis* DNA in surface water samples from different locations of the Chesapeake Bay and the Ohio Canal (C & O Canal) in Maryland, USA [77], which were later confirmed by two molecular methods [82]. Of the six samples (10 L) collected at the canal, 50% were considered positive using qPCR targeting the *C. cayetanensis* 18S rRNA gene, and a conventional PCR targeting the *C. cayetanensis* mitochondrial cytochrome oxidase gene and Sanger sequencing [82]. The DNA sequencing produced sequences that were 100% like other *C. cayetanensis* mitochondrial genome sequences.

Another recent study comparing filtration techniques used for the detection of *C. cayetanensis* in either produce irrigation or wash water found that DEUF was more robust than the USEPA Method 1623.1 for oocyst recovery from irrigation water (median *C. cayetanensis* recovery efficiencies were 17% for DEUF and 16–22% for Method 1623), while continuous flow centrifugation (CFC), which was evaluated separately, was demonstrated to be suitable for the recovery of *C. cayetanensis* oocysts from environmental and produce wash water [80]. Median *C. cayetanensis* recovery efficiencies for CFC were 28% for wash water and 63% for creek water, making it a viable option for processing water with high turbidity or organic matter. In this same study, it was noted that DEUF filters were able to filter larger volumes of turbid water than the filters used in USEPA Method 1623.1. Increased filtration volume may be beneficial for processing water samples from diverse sources, which would assist in understanding the sources of *C. cayetanensis* in the environment [80].

### 5.3. Soil

Analysis of soil samples will be useful in identifying environmental sources of *C. cayetanensis* contamination, the persistence of the parasite in the soil, and the role of soil in the transmission of *C. cayetanensis*, but studies in soil are still limited. The same limitations pointed out for water studies are even greater for the detection of the parasite in soil. It is expected that the infectious stage of the parasite may be present in soil at very low concentrations, and soil is a very complex substrate that may contain a variety of contaminating substances. As with water, the detection of *C. cayetanensis* in soil may require initial wash and concentration techniques that enrich and purify the oocyst form of the parasite to enhance detection, although some studies have performed DNA isolation directly from soil samples [64]. Testing for the presence of *C. cayetanensis* is later performed using microscopy, molecular methods, or both. The techniques for *C. cayetanensis* detection in soil must also be both sensitive and specific, as soil may contain closely related organisms that are not human pathogens.

In the two recent studies that reported the presence of *C. cayetanensis* in soil, different methods for sample preparation and detection of the parasite were used. Wash and concentration steps followed by microscopic observation of oocysts were used in one study [22], while a second study performed DNA extraction from approximately 250 mg of each soil sample and molecular detection was performed by nested PCR of the 18S rRNA. The PCR products were then subjected to Sanger sequencing and phylogenetic analysis [64].

Studies with the expressed goal of developing detection methods for *C. cayetanensis* specifically for soil are rare. Only one recent study described a method for the concentration of oocysts and molecular detection of *C. cayetanensis* in soil samples [97]. The study evaluated a concentration method using flotation in saturated sucrose solution and compared it to three commercial DNA isolation kits in experimentally seeded *C. cayetanensis* soil samples (5–10 g). Significantly lower cycle threshold values (C_T_) were observed in the *C. cayetanensis* samples processed via the flotation method than those processed with each of the commercial DNA isolation kits evaluated (*p <* 0.05). Linearity of detection of the flotation method was observed and it was able to detect as few as 10 oocysts in 10 g of soil samples. This comparative study showed that the concentration of oocysts in soil samples by flotation in high-density sucrose solutions is an easy, low-cost, and sensitive method that could be implemented for the detection of *C. cayetanensis* in environmental soil samples [97]. The flotation method was able to detect low numbers of oocysts in two different types of farm soil (10 oocysts in 10 g of either type of farm soil) without modifications. When processing commercial potting mix, the method needed an extra wash to eliminate particulate matter, and reduced size samples to be able to detect 20 oocysts/5 g [98].

### 5.4. Clinical Samples

Cyclosporiasis is still not often considered by healthcare providers, and many clinical laboratories do not routinely perform testing for this parasite. At clinical laboratories, diagnosis of cyclosporiasis still relies heavily on microscopy [99]. Detection of the spherical *C. cayetanensis* oocysts (8–10 µm in diameter) in stool samples is challenging, and if only a routine ova-and-parasite exam is conducted, the presence of *C. cayetanensis* may go unnoticed. As with other intestinal parasites, the collection of multiple stool samples is highly recommended to achieve better detection as the number of oocysts in stool may be low [99]. Enhanced detection using differential interference contract (DIC) or ultraviolet (UV) autofluorescence is recommended; however, they are not always available in clinical laboratories. Currently, there are no antibody or antigen detection assays for routine diagnosis of cyclosporiasis in clinical samples.

Multiplex molecular assays have the benefit of allowing comprehensive testing for gastrointestinal pathogens that in most cases have overlapping clinical presentations, with fast results and high-throughput testing. Currently, five commercially multiplex molecular assays for gastrointestinal pathogen detection that include *C. cayetanensis* are currently available (BioFire^®^ FilmArray^®^ Gastrointestinal (GI) Panel (Biomerieux, Inc. Salt Lake City, UT, USA), Allplex^TM^ Gastrointestinal Panel (Seegene USA, Inc. Irvine, CA, USA), QIAstat-Dx^®^ Gastrointestinal Panel (Qiagen, Germantown, MD, USA), EasyScreen^TM^ Enteric Protozoan Extended Detection Kit (Genetic Signatures Ltd. Newtown, Australia), and Novadiag^®^ Stool parasites (Hologic Inc. Malborough, MA, USA). Only the BioFire^®^ FilmArray^®^ Gastrointestinal (GI) Panel is approved by the FDA, whereas all five are approved by the European Union [99]. The FDA-approved BioFire^®^ FilmArray^®^ Gastrointestinal (GI) Panel enables rapid and accurate automated testing for 22 common gastrointestinal pathogens, including viruses, bacteria, and parasites. Among the five parasites included in the panel is *C. cayetanensis*. The BioFire^®^ FilmArray^®^ Gastrointestinal (GI) Panel was reported to be critical in the early recognition of a cyclosporiasis outbreak that occurred in Wisconsin in 2018 [100]. In fact, most of the initial *Cyclospora*-positive specimens of the outbreak were detected using the assay, indicating that these types of assays could assist in early outbreak recognition.

During an outbreak, the possibility of trace-back is critical by linking cases to each other as well as to a particular food source of infection. Whole-genome sequencing has become a routine practice to support epidemiological investigation of bacterial-caused foodborne outbreaks [101,102]; however, it is currently impractical for a routine genotyping approach for cyclosporiasis due to constraints in obtaining enough DNA from clinical isolates. Additionally, genotyping *C. cayetanensis* has the additional challenge of having samples with potentially high genetic heterogeneity associated with its sexual reproductive cycle. Currently, for cyclosporiasis, genotyping relies on amplification and sequencing markers that have polymorphism or variability in repeat regions. There are several molecular typing tools for cyclosporiasis that have been established to assist with epidemiological investigations of outbreaks [103,104]. The first described genotyping tool for *C. cayetanensis* used a Multilocus Sequence Typing (MLST) method that included five microsatellite loci (CYC3, CYC13, CYC15, CYC21, and CYC22), which was able to identify geographic differences among the 34 isolates included in the study [105]. An adaptation of this early method was recently evaluated. The modification included the omission of nested PCR for target amplification (inner primers were used for the single-step PCR) and the inclusion of four markers (CYC3, CYC13, CYC21, and CYC22); marker CYC15 was not included due to the limited variability observed among isolates [103]. This evaluation using *Cyclospora*-positive stool specimens from 54 patients noted that markers CYC3 and CYC13 frequently generated unreadable DNA sequences and they were excluded for further evaluation. Markers CYC21 and CYC22 showed better typeability power (ability to assign a type to a specimen) (84%) but lower discriminatory power (ability to assign a different type to unrelated specimens) (89%) than the original method. In another study conducted to identify additional markers able to assist with genotyping, two nuclear (HC378 and HC360i2) and one mitochondrial (MSR) SNP-rich loci were evaluated and showed a 90% amplification and sequencing success rate [106]. Eighty-eight specimens were subjected to PCR and Sanger sequencing of those three loci; following genetic clustering, 16 clusters were identified with epidemiological data supporting the clustering, suggesting that markers could be helpful in assisting with epidemiological investigations of cyclosporiasis outbreaks [106]. Additional markers suitable for genotyping clinical samples were still necessary to improve capturing genetic variability among cyclosporiasis cases were still required. A workflow to identify additional SNPs in the nuclear genome to increase genotyping markers for *C. cayetanensis* using whole-genome comparison of four isolates was conducted [103]. The search identified four markers (CDS-1, CDS-2, CDS-3, and CDS-4) that were tested using 93 specimens, with 84, 83, 73, and 78 specimens employed to test CDS-1, CDS-2, CDS-3, and CDS-4, respectively. Amplification of all four markers was only possible in 57 specimens, and individual success rates were 61%, 77%, 75%, and 74 for CDS-1, CDS-2, CDS-3, and CDS-4, respectively. Using those four markers, 19 unique genotypes were identified among the 57 specimens.

The mitochondrial genome is a suitable target for genotyping; it evolves faster than nuclear genomes, providing high resolution, and the high copy numbers of the mitochondrial genome per cell for *C. cayetanensis* assist with sensitivity [107]. A quantitative PCR targeting a polymorphic link region of the mitochondrial genome to evaluate genetic heterogenicity among *C. cayetanensis* isolates using melt curves and gel electrophoresis analysis revealed significant geographic segregation among 36 clinical specimens that included samples from six countries [108]. Sequencing of the same polymorphic link region of the mitochondrial genome was used to genotype clinical stool samples from 134 laboratory-confirmed cases of cyclosporiasis [109]. Although the assay was able to identify 14 genotypes and the genotypes identified were identical among samples for all linked cases within 7 of the 10 clusters, it was not enough to discriminate all outbreak clusters. A method to generate the complete mitochondrial genome of *C. cayetanensis* to capture the diversity of the parasite was used in clinical samples with different levels of positivity (based on qPCR CT values) [93]. Phylogenetic analysis of the mitochondrial genome profiles showed grouping with different geographical origins. It was proposed that mitochondrial genomes generated should be uploaded on the NCBI CycloTrakr database to allow comparisons of genomes obtained from future outbreaks.

Next-generation amplicon sequencing and ensemble-based distance statistic that allows for mixed genotypes and specimens with partial genotype data were used to evaluate the genotyping of 648 *C. cayetanensis* samples submitted to CDC in 2018 for US cyclosporiasis outbreaks [110]. The protocol included eight markers previously evaluated (CDS-1, CDS-2, CDS-3, CDS-4, HC378, HC360i2, Mt-junction, and MSR) [103,106,109], but used next-generation amplicon sequencing instead of Sanger sequencing to allow capturing haplotype diversity. The approach allowed for genetic clustering with 93.8% and 99.7% sensitivity and specificity, respectively, and it showed that it could be used to investigate outbreaks. However, it was noted that the sequencing success varied from 53.2% to 97.9% among the eight markers used [110], and missing data were common in the results. In fact, only 34.4% of specimens included in the analysis generated data for all markers, and 13.1% of samples were genotyped at only four of the eight markers [110]

The US Centers for Disease Control and Prevention (CDC) has developed a *C. cayetanensis* genotyping system known CYCLONE (CYbernetic CLustering Of Non-clonal Eukaryotes). CYCLONE involves deep-sequencing using Illumina MiSeq platform of amplicons generated by PCR amplification of eight markers, six from the nuclear genome (CDS1, CDS2, CDS3, CDS4, 360i2, and 378) and two from the mitochondrial genome (Mt junction and MSR), previously described for genotyping purposes for *C. cayetanensis* [103,106,109,110]. CYCLONE Bioinformatic analysis includes multiple steps including identifying genotypes based on haplotypes for each marker, calculating pairwise genetic distances using an ensemble learning method, and clustering of genetic distance for downstream analysis [109,111,112].

A newly developed targeted amplicon sequencing (TAS) assay targeting 52 loci (49 of those in the nuclear genome and the rest at the mitochondrial level), encompassing 396 currently known SNP sites for genotyping *C. cayetanensis*, was recently reported [95]. Clinical fecal samples with low parasite loads were successfully genotyped. This method will greatly expand the genomic diversity included for genetic clustering of clinical specimens and has also been successful in genotyping produce samples with low levels of *C. cayetanensis* oocysts [95].

## 6. Pathogenesis, Symptoms, and Treatment

Several reviews on the clinical presentation, pathology, clinical diagnosis, and treatment of cyclosporiasis were recently published [21,99,113]. Review [113] included an in-depth discussion of current laboratory diagnostic methods for clinical cases of *C. cayetanensis* [96]. To our knowledge, there is no additional new information on the pathogenesis of infection within the past three years.

Cyclosporiasis was reported in 3 (3.44%) patients by microscopic examination of 87 patients with colorectal cancers in hospitals of Lorestan, Iran [114]. Some of the possible sequelae related to cyclosporiasis include Guillain–Barré syndrome and reactive arthritis [2]. In a recent study, a large percentage of patients (46.7%, 50/107) with unexplained rheumatic pain had a parasitic infection; cyclosporiasis was observed in 32% of those patients [115]. It has also been hypothesized that coccidia infections, including cyclosporiasis, could be environmental triggers for celiac disease [116]. Most reported clinical cases of cyclosporiasis in the past three years involved immunocompromised persons, such as HIV-positive, transplant recipients, or cancer patients [15,114,117,118,119,120]. A clinical case in an immunocompetent person was also reported [17].

To our knowledge, no new treatments have been used in humans in recent years. In recent clinical cases, patients were treated with trimethoprim/sulfamethoxazole [117,119]. The clinical cases included a patient with severe combined pulmonary *Nocardia* bacteremia with cyclosporiasis and a previous history of heart transplant in India [117] and an immunocompromised patient with large B cell lymphoma in Northern Spain [119]. Cotrimoxazole was successful in the treatment of an immunocompromised patient, who had previously undergone a renal allograft transplant and had a history of several coinfections (tuberculosis, cytomegalovirus, severe acute respiratory syndrome coronavirus 2, and hepatitis C) [120]. Ciprofloxacin, together with parenteral solutions, gastric mucosal protector, and soft diet was administrated as a successful treatment in a travel-related case in a kidney transplant patient who traveled from Mexico to The Netherlands [101].

## 7. Prevention and Control

The main prevention and control measures used for *C. cayetanesis* were previously summarized [2]; these included previously reported exploratory methods to remove or inactivate oocysts in fresh fruits and raw vegetables. To emphasize the need for prevention and control measures to reduce *C. cayetanesis* infections, a *Cyclospora* prevention, response, and research action plan was initially released in 2021 by the FDA in the US and has been recently updated [121]. In this multi-year strategic guide, there are three priority areas: improving prevention, enhancing response activities, and filling knowledge gaps. The plan included the main prevention and control measures for sources of contamination in the field, in the packinghouse, and among farm workers, such as the need for proper worker hygiene, workplace sanitation, and monitoring of inputs that may be contaminated by human feces (e.g., surface waters that may be impacted by sewage leaks), and included some measures to minimize the chance of contaminating fresh produce with *C. cayetanensis* [121].

There is a relationship between cyclosporiasis with poverty that carries implications for targeted public health interventions in resource-poor countries. Longitudinal and spatial analysis will be crucial to ascertain the impact of poverty on cyclosporiasis and the potential role of soil as a reservoir of infection to guide its prevention and control in endemic areas [122].

Some recent work has illustrated the potential and limits of using related organisms as surrogates to study the control of *C. cayetanensis*. Protozoa parasites such as *Eimeria* could be useful surrogates in exploratory studies because their oocysts are easier to acquire than those of *C. cayetanensis*. Additionally, many *Eimeria* species pose no risk to the health of human investigators. Thus, surrogates could speed up efforts to evaluate methods to filter parasites from irrigation water, treat food in ways that may render contaminants harmless, or treat infections when prevention fails [13,15,123]. Surrogates could also be studied in their natural hosts to evaluate whether an intervention rendered oocysts incapable of infecting their hosts.

## 8. Future Research

Currently, rapid test kits are not available to specifically detect *C. cayetanensis*. Developing specific rapid test kits for *C. cayetanensis* would allow for expanded testing and to conduct root cause analyses to assess potential sources and routes of contamination of *C. cayetanensis.*

For clinical cases, there are now genotyping methods and improved molecular detection methods for *C. cayetanensis*. In fresh produce, water, and soil, however, data on genetic characterization are still lacking, although new methods have been recently reported. This is due in part to the fact that methods used for clinical samples cannot be directly translated for use with environmental samples. The number of oocysts present in human samples may be many times higher than the low numbers of non-homogenously disseminated oocysts that are found in produce or the environment. Therefore, new methods and trace-back applications designed to address the characteristics of specific substrates are needed.

In the US, surveillance sampling of domestic and imported produce is needed to understand trends related to *C. cayetanensis* contamination, such as seasonality or geography. Sampling will also assist in identifying potential produce vehicles associated with outbreaks.

More research is needed to improve food safety and prevent outbreaks as well as to understand the life cycle stages of *C. cayetanensis* that take place in the environment. Future research efforts should focus on studies of the fate and transport of *Cyclospora* oocysts through soil and water, prevalence, factors associated with *C. cayetanensis* contamination of soil, and methods for controlling *Cyclospora* in the environment. The development of aptamers or immunomagnetic beads might help to isolate *C. cayetanensis* oocysts from environmental samples [124]. Similarly, there is a need to study oocysts in greater detail since proper TEM descriptions of *C. cayetanensis* oocysts are not available.

To understand seasonality, simultaneous studies from multiple countries utilizing the same sampling and testing are needed. However, the resources and degree of coordination required to conduct such a geographically and temporally large-scale project make such studies difficult to perform.

The development of viability methods is needed to test parasite mitigation and control measures and to study the persistence of *C. cayetanensis* in the environment. Work with a potential *C. cayetanensis* surrogate has provided some recent insight into changes in gene expression during oocyst development. It was reported that during the maturation of oocysts of *Eimeria acervulina* (a common poultry parasite), there is upregulation of a suite of genes, most of which have homologs in *C. cayetanensis* [123]. Such data may have potential as a first step towards the development of viability tests for *C. cayetanensis*.

## 9. Conclusions

In the past three years, some important advances in the life cycle, taxonomy, epidemiological worldwide data, risk factors, and particularly in new methodologies for the genotyping of clinical samples and the detection of *C. cayetanensis* in food and environment have been achieved. However, many unknowns remain to be explored. Further research is needed particularly for trace-back investigations in food and the environment to provide links between clinical cases and outbreaks, but also to establish measures of parasite viability and to determine the conditions under which sporulation takes place in the environment. Fortunately, some of these studies are currently underway.

## Data Availability

Not applicable.
